# Preoperative Prediction of Microvascular Invasion Risk Grades in Hepatocellular Carcinoma Based on Tumor and Peritumor Dual-Region Radiomics Signatures

**DOI:** 10.3389/fonc.2022.853336

**Published:** 2022-03-22

**Authors:** Fang Hu, Yuhan Zhang, Man Li, Chen Liu, Handan Zhang, Xiaoming Li, Sanyuan Liu, Xiaofei Hu, Jian Wang

**Affiliations:** ^1^ Department of Radiology, Southwest Hospital, Third Military Medical University (Army Medical University), Chongqing, China; ^2^ Department of Radiology, Tongliang District People's Hospital, Chongqing, China; ^3^ Department of Research and Development, Shanghai United Imaging Intelligence Co., Ltd., Shanghai, China

**Keywords:** hepatocellular carcinoma, microvascular invasion, radiomics, MRI, peritumor

## Abstract

**Objective:**

To predict preoperative microvascular invasion (MVI) risk grade by analyzing the radiomics signatures of tumors and peritumors on enhanced magnetic resonance imaging (MRI) images of hepatocellular carcinoma (HCC).

**Methods:**

A total of 501 HCC patients (training cohort n = 402, testing cohort n = 99) who underwent preoperative Gd-EOB-DTPA-enhanced MRI and curative liver resection within a month were studied retrospectively. Radiomics signatures were selected using the least absolute shrinkage and selection operator (Lasso) algorithm. Unimodal radiomics models based on tumors and peritumors (10mm or 20mm) were established using the Logistic algorithm, using plain T1WI, arterial phase (AP), portal venous phase (PVP), and hepatobiliary phase (HBP) images. Multimodal radiomics models based on different regions of interest (ROIs) were established using a combinatorial modeling approach. Moreover, we merged radiomics signatures and clinico-radiological features to build unimodal and multimodal clinical radiomics models.

**Results:**

In the testing cohort, the AUC of the dual-region (tumor & peritumor 20 mm)radiomics model and single-region (tumor) radiomics model were 0.741 vs 0.694, 0.733 vs 0.725, 0.667 vs 0.710, and 0.559 vs 0.677, respectively, according to AP, PVP, T1WI, and HBP images. The AUC of the final clinical radiomics model based on tumor and peritumoral 20mm incorporating radiomics features in AP&PVP&T1WI images for predicting MVI classification in the training and testing cohorts were 0.962 and 0.852, respectively.

**Conclusion:**

The radiomics signatures of the dual regions for tumor and peritumor on AP and PVP images are of significance to predict MVI.

## 1 Introduction

Hepatocellular carcinoma (HCC) has a high recurrence rate, with a five-year recurrence rate of 70% and 35% after liver resection and liver transplantation, respectively (1). Several findings suggest that microvascular invasion (MVI) is essential in the prognosis of HCC patients (2–4). Microvascular invasion is the formation of nested clusters of cancer cells in the lumen of endothelium-covered vessels on a microscopic scale (5), and it can only be detected using pathological diagnostics. In recent years, MVI has attracted increasing attention from clinicians, and the more severe the degree of microvascular invasion, the earlier the recurrence and the shorter the overall survival time of patients (6, 7). Zhao et al. (8) identified significant differences in the prognosis of HCC patients with different MVI risk grades. Furthermore, the cumulative five-year postoperative survival and tumor-free survival rates in the high-risk MVI group were only 25.4% and 15.8%, respectively, significantly worse than the low-risk MVI and no-MVI groups. Predicting preoperative MVI risk grading could help clinicians in providing personalized treatments to patients with high-risk HCC. Furthermore, several studies have illustrated that among HCC patients presenting with MVI, the anatomical liver resection group has a higher recurrence-free survival rate than the non-anatomical liver resection group (9, 10). This clearly demonstrates the importance of preoperative MVI prediction for improved prognosis of HCC patients.

Several scholars have attempted to predict MVI using hematologic indicators, such as serum alpha-fetoprotein (AFP) (11) or imaging features such as peritumoral hypointensity in the hepatobiliary phase (12, 13), arterial peritumoral enhancement (14), and nonsmooth tumor margins (12, 14) to find a reliable and non-invasive method for preoperative diagnosis of MVI. Although the results showed a correlation between AFP or these imaging features and MVI, the criteria for determining the AFP threshold value have not been identified. These imaging features lacked objectivity and were greatly influenced by the knowledge base, diagnostic experience, and work status of radiologists.

In recent years, with the advent of radiomics technology, some scholars have been extracting signatures from CT or MRI images that are difficult to perceive with human eyes, and building models to preoperatively predict negative or positive hepatocellular carcinoma MVI using automatic algorithms. Their findings demonstrate that radiomics signatures on radiological images are promising for preoperative prediction of MVI (15–28). Furthermore, attempts have been made to predict MVI preoperatively using tumor and peritumor radiomics signatures. Microvascular invasion is mostly common in small portal vein branches inside paracancerous liver tissue (29). However, whether peritumor signatures are valuable in predicting MVI is controversial.

Feng et al. (15) used Gd-EOB -DTPA-enhanced MRI radiomics signatures to predict MVI and found that peritumor signatures are important in MVI prediction. They also realized that the dual-region (tumor and peritumor; 10 mm) radiomics model was superior to the tumor radiomics model. In contrast, Xu et al. (24) found that the dual-region (tumor and peritumor; 5 mm) radiomics model did not highlight any advantages in predicting MVI compared to the tumor-based radiomics model. This discrepancy may be due to inconsistent imaging methods and peritumor extent. Therefore, to explore the impact of dual-region radiomics signatures of the tumor and peritumor on MVI prediction, this study aimed to develop enhanced MRI radiomics models with different ROIs (including tumor, tumor & peritumor 10 mm, and tumor & peritumor 20 mm) for preoperative prediction of MVI risk grades.

## 2 Methods

### 2.1 Patient Data Collection and Follow-Up

#### 2.1.1 Inclusion and Exclusion Criteria

We retrospectively analyzed a total of 501 HCC patients who met the inclusive criteria from June 2017 to July 2020. All patients were randomly divided two cohorts (4:1): a training cohort (n = 402) and a testing cohort (n = 99). The inclusion criteria included: (i) Gd-EOB-DTPA-enhanced MRI within one month before surgery; (ii) pathologically confirmed HCC; and (iii) curative surgical resection or liver transplantation. The exclusion criteria included: (i) history of recurrent HCC or HCC combined with other primary tumors; (ii) poor image quality; (iii) MRI showed large vessel cancer thrombus; and (iv) history of preoperative anti-cancer treatment. This study was approved by the ethical review committee of the First Affiliated Hospital of the Army Medical University. Patients were exempted from providing informed consent.

#### 2.1.2 Image Acquisition

Pre-scan preparation required the patients to fast and abstain from food and drinks for over six hours. Breathing training, which involved breath-holding in a calm state, was provided. A 3.0T MRI (magnetom trio, siemens healthcare, erlangen, Germany), 12-channel phased-array body coil, and high-pressure injector were used for image acquisition. The positioning image, in-phase and opposed-phase T1-weighted imaging (T1WI), and dynamic three-dimensional volumetric interpolated breath-hold examination (3D-VIBE) flat scan were obtained before MRI enhancement. Post-contrast dynamic 3D-VIBE was performed at the arterial phase (30 s), portal venous phase (70 s), transitional phase(3 min) and hepatobiliary phase(15min) after a rapid bolus injection of contrast agent (Primovist; Bayer Schering Pharma, Berlin, Germany) with a rate of 1 mL/s, followed by a 20 mL saline flush. T2-weighted images were obtained with a technique of half-Fourier acquisition single-shot fast spin-echo sequence. Diffusion-weighted imaging (DWI) adopts a breathing- triggered technique at b values of 0, 50, 400, and 800 s/mm^2^, and the apparent diffusion coefficient (ADC) was calculated using a single exponential function with b values of 0 and 800 s/mm^2^. Susceptibility weighted imaging (SWI) adopts high-resolution, 3D gradient echo and 3D fully flow-compensated sequence for scanning.

#### 2.1.3 Clinical and Imaging Data

Information including gender, age, cirrhosis, hepatitis B surface antigen (HBS Ag), platelet count (PLT), serum albumin (ALB), alanine transarninase (ALT), aspertate aminotransferase (AST), alkaline phosphatase (ALP), serum total bilirubin (TBIL), serum α-fetoprotein (AFP), activated partial thromboplastin time (APTT), prothrombin time (PT), international normalized ratio (INR) and other relevant details were collected from the electronic medical record system and laboratory tests. Patients were evaluated and classified according to MRI manifestations and laboratory tests using liver function grading criteria (Child-Pugh).

All MRI image features were analyzed jointly by two radiologists with three and four years of diagnostic abdominal imaging experience, respectively. Then the results were reviewed by two senior doctors and cross-reviewed. If there is any dispute, the final decision will be made after discussion by two doctors. If there is any dispute, the final decision will be made after discussion by two doctors. During the image analysis, the four aforementioned radiologists did not refer to clinical laboratory tests, other imaging tests, or postoperative pathological diagnoses. The assessment of imaging features included the number of tumors, the maximum length of the tumor, satellite noduels, tumor morphology, tumor envelope integrity, intra-tumor hemorrhage, intra-tumor fat, arterial peritumor enhancement, and hepatobiliary peritumor hypointensty. The maximum length of the tumor is measured in the coronal, sagittal, or axial plane. The capsule is defined as the portal venous phase or delayed phase, with annular high enhancement around the lesion (30). The state of the capsule is divided into two types: intact, incomplete or absent. Satellite noduels mainly refers to the small tumor focus with a diameter ≤ 2cm within the range of the main tumor ≤ 2cm (5). The shape of the tumor was evaluated as round or irregular. Intratumoral hemorrhage defined as low signal intensity in SWI phase, intratumoral fat defined as high signal intensity in the in- phase and low signal intensity in the opposed-phase. Peritumoral enhancement in arterial phase defined as obvious crescent or patchy enhancement in arterial phase, but consistent with hepatic parenchyma enhancement in portal venous phase (31). Peritumoral hypointensity in the hepatobiliary phase is defined as patchy abnormal signal shadow around hepatobiliary tumor, and the signal intensity is lower than that of normal liver parenchyma (32).

#### 2.1.4 Evaluation of Pathological MVI

Two pathologists assessed the MVI status of all HCC cases by examining the hematoxylin-eosin (HE) stained sections under a microscope. The Guidelines for the Standardized Pathological Diagnosis of Primary Liver Cancer (2015 edition) were used to grade MVI risk. The three risk levels of MVI included M0: no MVI detected; M1 (low-risk group): 0<the number of MVI ≤ 5 and MVI occurred in the proximal paracancerous liver(<1cm); M2 (high-risk group): the number of MVI > 5 MVI or MVI occurred in the distal paracancerous liver tissue area (> 1cm) (29).

#### 2.1.5 Follow-Up Visits

The endpoint of this study, which was December 31, 2020, was considered the date of recurrence. The time from the first postoperative day to tumor recurrence or termination for follow-up observation was referred to as recurrence-free survival (RFS). Postoperative recurrence was mainly detected using CT, MRI, ultrasonography and other imaging examinations, combining with laboratory examinations, such as serum AFP at the same time. The time of recurrence was recorded after the diagnosis of recurrence.

### 2.2 Enhanced MRI Radiomics Analysis

**Figure 1:**
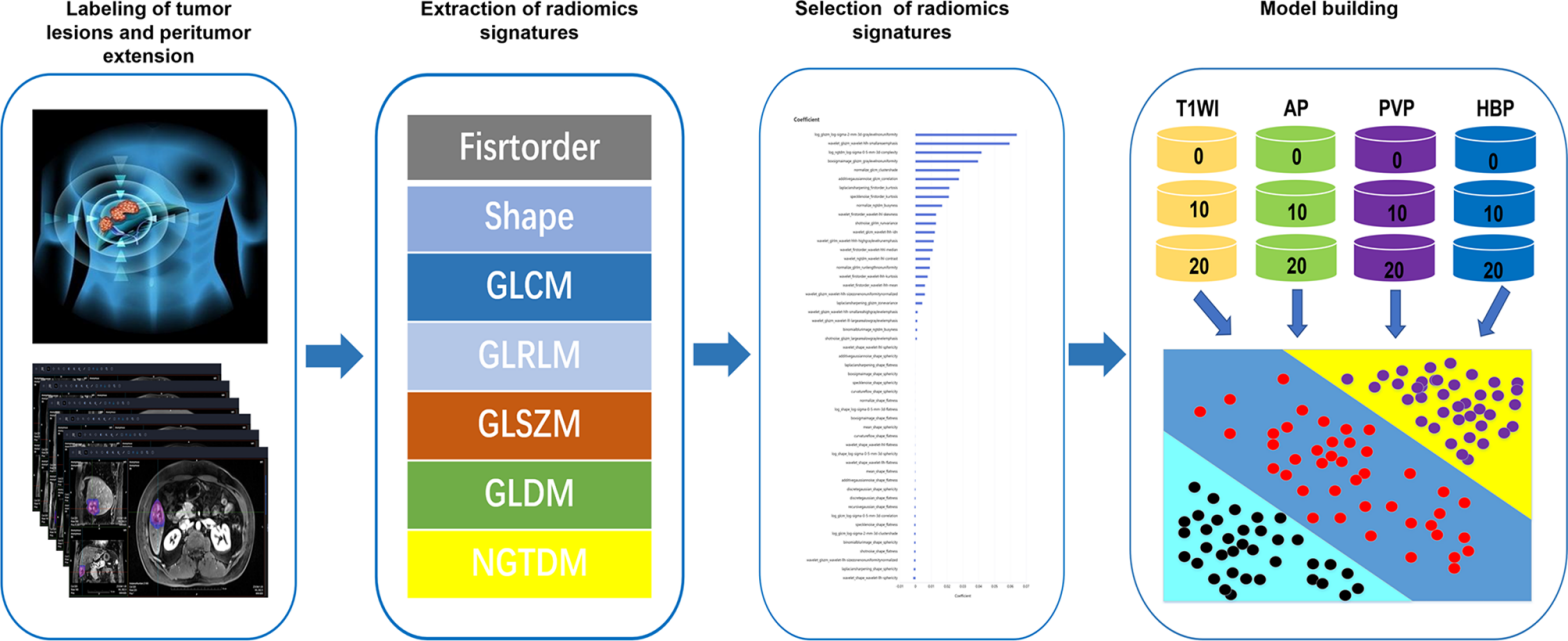
Workflow of radiomics analysis. The radiomics workflow started with labeling of tumor lesions and peritumor extension in MR images. After that, radiomic features including firstorder, shape, gray-level co-occurrence matrix (GLCM), gray-Level run-length matrix (GLRLM), gray-level size zone matrix (GLSZM), gray level difference method (GLDM) and neighborhood gray-tone difference matrix (NGTDM) were extracted within the tumor and peritumor dual-region. Next, least absolute shrinkage and selection operator (LASSO) were used for the radiomic feature selection. Finally, unimodal radiomics models and multimodal radiomics models based on different regions of interest (ROIs) in the tumor and peritumor was developed.

Radiomics analyses were performed at uAl-Research-Portal(Shanghai United Imaging Intelligence Co., Ltd), a clinical research platform written in the Python programming language (version 3.7.3, https://www.python.org). The widely used software package Py Radiomics (https://pyradiomics.readthedocs.io/en/latest/html) is embedded in this platform. Enhanced MRI radiomics analysis included annotation of tumor lesions and peritumor extension, extraction and selection of radiomics signatures, and model building (**Figure 1**).

#### 2.2.1 Labeling of Tumor Lesions and Peritumor Extension

**Figure 2:**
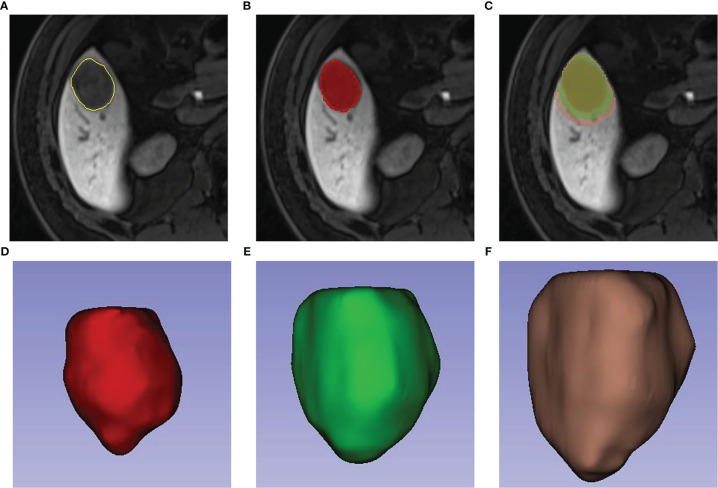
Labeling of tumor lesions and peritumor extension. First, radiologists manually draw the volume(VOI) of the tumor **(A, B)**. On the bases of VOI entire of the tumor, a region with 10-mm and 20-mm distance to tumor surface were automatically reconstructed **(C)**. 3-dimensional view of the VOI entire, VOI (tumor & peritumor 10 mm) and VOI (tumor & peritumor 20 mm) respectively were showed in Picture **(D–F)**.

Two physicians with three and four years of diagnostic abdominal MRI experience, respectively, selected plain T1WI, arterial phase (AP), portal venous phase (PVP), and hepatobiliary phase (HBP) sequences to label tumor lesions on a 3D slicer. The annotation results of all tumor lesions were validated by two radiologists with nine years of experience in diagnostic abdominal imaging. The annotation of 304 MRI liver data was completed for T1WI, resulting in a preliminary version of the automated liver segmentation model for T1WI. The T1WI liver segmentation was also extended to AP, PVP, and HBP images using the alignment and fine-tuning. Furthermore, the original tumor lesions were extended by 10 mm and 20 mm, respectively, in the uAl-Research-Portal (Shanghai United Imaging Intelligent Medical Technology Co., Ltd.). The extension beyond the boundary of the liver was adjusted by combining the results of the liver segmentation model (**Figure 2**).

#### 2.2.2 Extraction and Selection of Radiomics Signatures

The images were imported into uAl-Research-Portal (Shanghai United Imaging Intelligent Medical Technology Co., Ltd.) and preprocessed by resampling all voxels of images to 1 × 1 × 1 mm^3^ using the 3D nearest neighbor interpolation method. Then, 2,600 radiomics signatures were extracted within the lesion annotation range, respectively from T1WI of different ROI (tumor edge, tumor and peritumor 10 mm, and tumor and peritumor 20 mm), AP, PVP, and HBP modalities. In the training cohort, signatures were selected using the least absolute shrinkage and selection operator (Lasso) algorithm. The results of multiple ROI signatures selection with the same modality were merged to eliminate the possible influence of distinct radiomics types of signatures after signatures selection. We selected 85, 185, 178, and 62 radiomics signatures as the most critical for MVI risk grading on T1WI, AP, PVP, and HBP, respectively.

### 2.3 Model Building

#### 2.3.1 Building Unimodal Radiomics Models Based on Different ROIs in the Tumor and Peritumor

Box-Cox transformation was performed on radiomics signatures after selecting T1WI, AP, PVP, and HBP modal features. According to above phases, Logistic algorithm was used to build unimodal radiomics models based on tumor, tumor and peritumor 10 mm, and tumor and peritumor 20 mm [T1WI (0\10\20), AP (0\10\20), PVP (0\10\20), HBP (0\10\20)] in the training cohort. In addition, their ability to predict MVI was tested in the testing cohort.

#### 2.3.2 Building Multimodal Radiomics Models Based on Different ROIs in the Tumor and Peritumor

Tumor-based unimodal radiomics models were combined to create an unexpanded multimodal peritumor radiomics model. The prediction probabilities of each modality corresponding to the MVI category were summed to determine the final prediction sequence. The best tumor-based radiomics model for ROI was selected based on the AUC. The combination modeling method described above was then used to create multimodal radiomics models with different ROIs for tumor and peritumor (10 mm and 20 mm) accordingly.

#### 2.3.3 Building Clinical Radiomic Model

The radiomic model with the best predictive performance for MVI risk grading was selected based on the AUC. We combined the essential clinical and radiological features selected using the Lasso algorithm with the corresponding unimodal radiomics signatures in the best radiomics model to build a unimodal clinical radiomics model. We also established a multimodal clinical radiomics model using the above-combined modeling approach.

### 2.4 Statistical Analysis

Statistical analysis was performed using R software. The rank sum test, one-way ANOVA, and chi-square test were used to analyze statistical differences between clinical indicators and radiological signals in M0, M1, and M2 groups. The ROC curves of different models were plotted, while AUC values were calculated using PyCharm software. Survival curves were plotted using Kaplan-Meier and tested using the two-sided log-rank test. A two-tailed *p* value less than 0.05 was considered statistically significant.

## 3 Results

### 3.1 Clinical and Imaging Features of Patients

**Figure 3:**
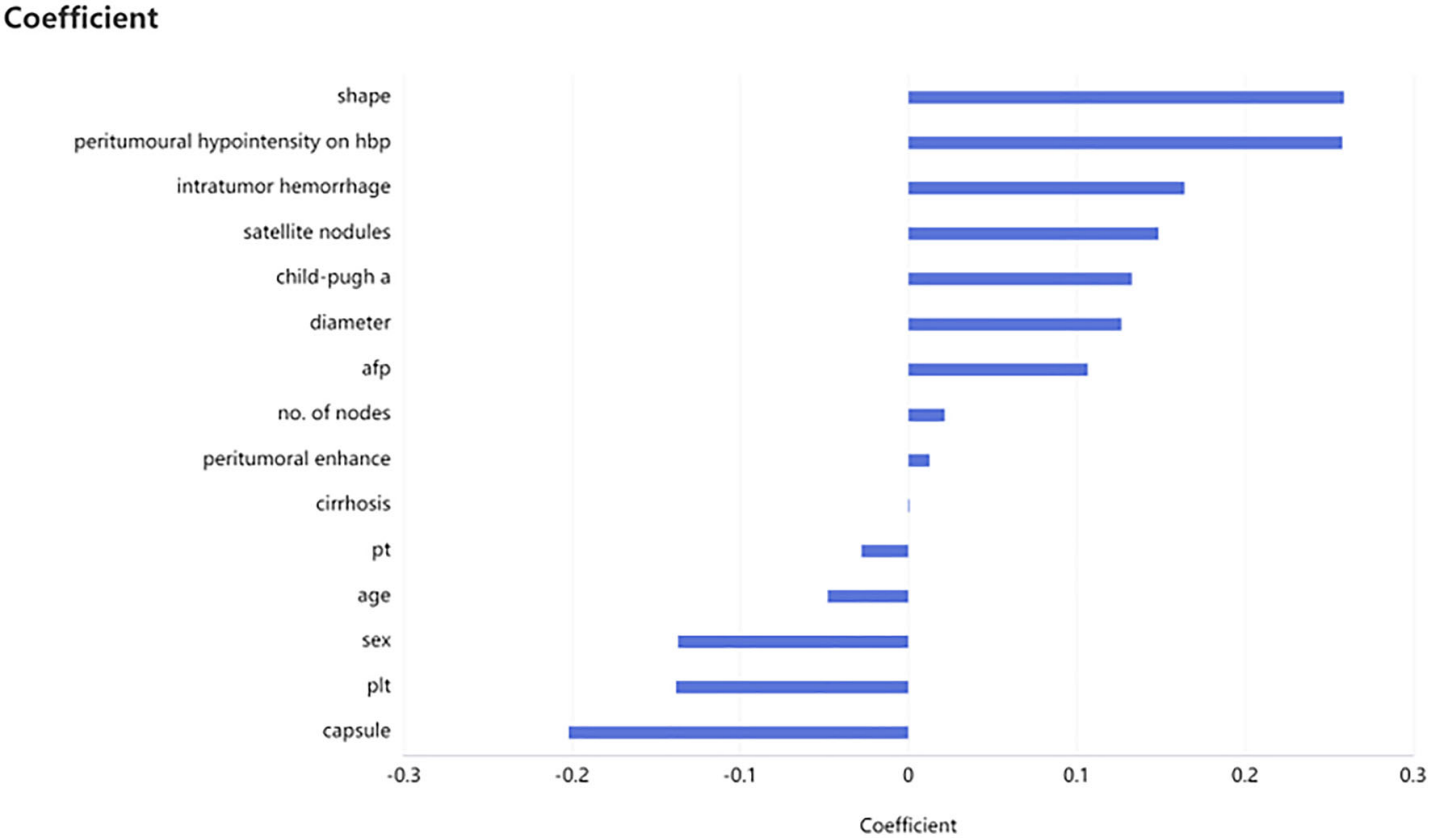
Selection of clinicoradiological characteristics. Fifteen necessary clinicoradiological features were finally selected by using least absolute shrinkage and selection operator (LASSO), including serum AFP level, Child-Pugh, cirrhosis, age et al.

A total of 501 patients met the inclusive and exclusive criteria, with 252 (50.3%) patients were pathologically diagnosed as MVI negative and 249 (49.70%) patients were pathologically identified as MVI positive: 207 (41.32%) in group M1 and 42 (8.38%) in group M2. The three groups were statistically different in INR, AFP, Child-Pugh, number of nodes, shape, arterial peritumoral enhancement, peritumoral hypointensity in the hepatobiliary phase, tumor diameter, intratumoral hemorrhage, satellite foci, and envelope (*p* < 0.05), but not in the remaining clinical and radiological indices (**Table 1**). The differences of clinicoradiological characteristics in between training and testing datasets are listed in **Table 2**. A total of 24 clinical and radiological signatures were selected using the Lasso algorithm. Fifteen essential clinical and radiological features, including serum AFP level, Child-Pugh, cirrhosis, age, PT, PLT, shape, peritumoral hypointensity in the hepatobiliary phase, intratumoral hemorrhage, satellite foci, diameter, number of nodes, arterial peritumoral enhancement, envelope, and tumor diameter, were finally selected (**Figure 3**).

**Table 1:** Comparisons of clinicoradiological characteristics in different microvascular invasion grades.

Variable	M 0n =252	M1n =207	M2n =42	P-value
Age (years)	52.81 ± 10.56	50.99 ± 11.22	51.12 ± 11.37	0.183
Gender				0.344
male	210 (83.33%)	180 (86.96%)	38 (90.48%)	
female	42 (16.67%)	27 (13.04%)	4 (9.52%)	
PLT, 10^9/L	150.25 ± 74.36	157.60 ± 71.89	158.79 ± 91.23	0.528
ALT, IU/L	62.15 ± 90.40	63.92 ± 97.84	80.62 ± 110.65	0.507
AST, IU/L	56.57 ± 77.51	63.22 ± 92.43	65.45 ± 47.28	0.625
ALB, g/L	41.80 ± 5.04	41.97 ± 5.02	41.69 ± 8.20	0.922
TBIL, μmol	18.39 ± 20.98	18.08 ± 9.57	18.33 ± 6.67	0.979
ALP, IU/L	113.45 ± 96.19	109.72 ± 67.12	123.58 ± 86.38	0.615
APTT, sec	25.91 ± 19.23	28.09 ± 2.82	28.37 ± 7.00	0.202
PT, sec	13.81 ± 12.57	11.91 ± 5.17	12.15 ± 1.18	0.092
INR	1.01 ± 0.11	1.00 ± 0.08	1.05 ± 0.11	0.02
AFP				0.005
0, normal	112 (45.90%)	70 (34.48%)	10 (23.81%)	
1, abnormal	132 (54.10%)	133 (65.52%)	32 (76.19%)	
Hbs Ag				0.315
0, Hbs Ag(-)	38 (15.14%)	21 (10.34%)	5 (12.20%)	
1, Hbs Ag(+)	213 (84.86%)	182 (89.66%)	36 (87.80%)	
Child-Pugh				0.045
0, A	248 (98.41%)	203 (98.54%)	39 (92.86%)	
1, B	4 (1.59%)	3 (1.46%)	3 (7.14%)	
Cirrhosis				0.977
0, no	68 (26.98%)	56 (27.32%)	12 (28.57%)	
1, yes	184 (73.02%)	149 (72.68%)	30 (71.43%)	
No. of nodes				0.021
0, 1	242 (96.03%)	190 (91.79%)	36 (85.71%)	
1, ≥ 2	10 (3.97%)	17 (8.21%)	6 (14.29%)	
Shape				<0.001
0, circle	151 (59.92%)	52 (25.12%)	5 (11.90%)	
1, irregular	101 (40.08%)	155 (74.88%)	37 (88.10%)	
Arterial peritumoral enhancement				<0.001
0, absent	211 (83.73%)	136 (65.70%)	23 (54.76%)	
1, present	41 (16.27%)	71 (34.30%)	19 (45.24%)	
Peritumoral hypotensity on HBP				<0.001
0, absent	219 (86.90%)	145 (70.05%)	16 (38.10%)	
1, present	33 (13.10%)	62 (29.95%)	26 (61.90%)	
The maximum length				<0.001
0, ≤5cm	200 (79.37%)	125 (60.39%)	16 (38.10%)	
1, >5cm	52 (20.63%)	82 (39.61%)	26 (61.90%)	
Intratumoral hemorrhage				<0.001
0, absent	181 (71.83%)	113 (54.59%)	12 (28.57%)	
1, present	71 (28.17%)	94 (45.41%)	30 (71.43%)	
Intratumoral fat				0.204
0, absent	214 (84.92%)	180 (86.96%)	32 (76.19%)	
1, present	38 (15.08%)	27 (13.04%)	10 (23.81%)	
Satellite nodules				<0.001
0, absent	241 (96.02%)	186 (89.86%)	34 (80.95%)	
1, present	10 (3.98%)	21 (10.14%)	8 (19.05%)	
Capsule				<0.001
0, absence or incomplete	71 (28.17%)	136 (65.70%)	31 (73.81%)	
1, complete	181 (71.83%)	71 (34.30%)	11 (26.19%)	

Unless otherwise noted, data are shown as number of patients, with the percentage in parentheses. MVI, microvascular invasion.M 0= no MVI; M1=≤ 5 MVI, and occurred in the adjacent liver tissue area (≤ 1 cm); M2= > 5 MVI, or MVI occurred in the distant paracancerous liver tissue area (> 1cm).

PLT, platelet count; ALT, alanine transarninase; AST, aspertate aminotransferase; ALB, serum albumin; TBIL, serum total bilirubin; ALP, Alkaline phosphatase; APTT, activated partial thromboplastin time; PT, prothrombin time; INR, international normalized ratio; AFP, serum a-fetoprotein; Hbs Ag, hepatitis B surface antigen; HBP, hepatobiliary phase.

**Table 2:** The differences of clinicoradiological characteristics in between training and testing datasets.

Group	taining	testing	P-value
N	402	99	
Age (years)	51.66 ± 10.89	52.93 ± 11.03	0.301
PLT, 10^9/L	156.62 ± 77.43	143.45 ± 62.58	0.117
ALT, IU/L	59.16 ± 87.60	85.86 ± 119.67	0.012
AST, IU/L	55.04 ± 73.86	80.45 ± 107.22	0.006
ALB, g/L	41.96 ± 5.11	41.48 ± 6.26	0.422
TBIL, μmol	17.42 ± 8.33	21.64 ± 32.26	0.020
ALP, IU/L	112.84 ± 91.99	112.46 ± 41.64	0.969
APTT, sec	27.61 ± 14.69	24.60 ± 10.11	0.054
PT, sec	13.17 ± 10.65	11.75 ± 1.17	0.186
INR	1.00 ± 0.09	1.04 ± 0.14	<0.001
Gender			0.146
0, male	348 (86.57%)	80 (80.81%)	
1, female	54 (13.43%)	19 (19.19%)	
AFP			0.761
0, normal	153 (38.93%)	39 (40.62%)	
1, abnormal	240 (61.07%)	57 (59.38%)	
Hbs Ag			0.912
0, Hbs Ag(-)	51 (12.85%)	13 (13.27%)	
1, Hbs Ag(+)	346 (87.15%)	85 (86.73%)	
Child-Pugh			0.414
0, A	394 (98.25%)	96 (96.97%)	
1, B	7 (1.75%)	3 (3.03%)	
Cirrhosis			0.611
0, no	107 (26.75%)	29 (29.29%)	
1,yes	293 (73.25%)	70 (70.71%)	
No. of nodes			0.254
0, 1	373 (92.79%)	95 (95.96%)	
1, ≥ 2	29 (7.21%)	4 (4.04%)	
The maximum length			0.739
0,≤5cm	275 (68.41%)	66 (66.67%)	
1, >5cm	127 (31.59%)	33 (33.33%)	
Shape			0.179
0, circle	161 (40.05%)	47 (47.47%)	
1, irregular	241 (59.95%)	52 (52.53%)	
Satellite nodules			0.048
0, absent	365 (91.02%)	96 (96.97%)	
1, present	36 (8.98%)	3 (3.03%)	
Capsule			0.496
0, absence or incomplete	194 (48.26%)	44 (44.44%)	
1, complete	208 (51.74%)	55 (55.56%)	
Intratumoral hemorrhage			0.137
0, absent	252 (62.69%)	54 (54.55%)	
1, present	150 (37.31%)	45 (45.45%)	
Intratumoral fat			0.796
0, absent	341 (84.83%)	85 (85.86%)	
1, present	61 (15.17%)	14 (14.14%)	
Arterial peritumoral enhancement			0.294
0, absent	301 (74.88%)	69 (69.70%)	
1, present	101 (25.12%)	30 (30.30%)	
Peritumoral hypotensity on HBP			0.034
0, absent	313 (77.86%)	67 (67.68%)	
1, present	89 (22.14%)	32 (32.32%)	
MVI grade			0.993
0	202 (50.25%)	50 (50.51%)	
1	166 (41.29%)	41 (41.41%)	
2	34 (8.46%)	8 (8.08%)	

MVI, microvascular invasion; M0: no MVI detected; M1 (low-risk group): 0<the number of MVI ≤ 5 and MVI occurred in the proximal paracancerous liver; M2 (high-risk group): the number of MVI > 5 MVI or MVI occurred in the distal paracancerous liver tissue area (> 1 cm). PLT, platelet count; ALT, alanine transarninase; AST, aspertate aminotransferase; ALB, serum albumin; TBIL, serum total bilirubin; ALP, alkaline phosphatase; APTT, activated partial thromboplastin time; PT, prothrombin time; INR, international normalized ratio; AFP, serum afetoprotein; Hbs Ag, hepatitis B surface antigen; HBP, hepatobiliary phase

### 3.2 Building Models for Predicting MVI Risk Grading

#### 3.2.1 Developing Unimodal Radiomics Models Based on Different ROIs in the Tumor and Peritumor

The prediction results of unimodal radiomics models based on different ROIs in the tumor and peritumor are shown in **Table 3**. According to T1WI and HBP images, the radiomics model with ROI based on tumor [T1WI (0), HBP (0)] had the best prediction results. The AUC and ACC in the testing cohort were 0.710 and 0.677 and 0.566 and 0.535, respectively. However, the radiomics model with ROI based on tumor and peritumor 20mm on AP and PVP images [AP (20),PVP (20)] performed better in predicting MVI risk grades in the testing cohort in the unimodal radiomics models using different ROIs, with AUC of 0.741 and 0.733 and ACC of 0.556 and 0.586, respectively.

**Table 3:** Unimodal radiomics models based on different ROIs.

Modality	ROI	Training group		Testing group	
		AUC	ACC	AUC	ACC
T1WI	Tumor	0.796	0.644	0.710	0.566
	Tumor & Margin (10)	0.803	0.632	0.604	0.535
	Tumor & Margin (20)	0.780	0.617	0.667	0.566
AP	Tumor	0.893	0.726	0.694	0.505
	Tumor & Margin (10)	0.909	0.776	0.718	0.596
	Tumor & Margin (20)	0.927	0.808	0.741	0.556
PVP	Tumor	0.907	0.741	0.725	0.545
	Tumor & Margin (10)	0.911	0.766	0.726	0.586
	Tumor & Margin (20)	0.921	0.769	0.733	0.586
HBP	Tumor	0.795	0.657	0.677	0.535
	Tumor & Margin (10)	0.781	0.639	0.636	0.505
	Tumor & Margin (20)	0.777	0.617	0.559	0.424

#### 3.2.2 Building Multimodal Radiomics Models Based on Different ROIs in the Tumor and Peritumor

The prediction results of multimodal radiomics models using different ROIs of the tumor and peritumor are presented in **Table 4**. The fusion radiomics model [T1WI (0) &  PVP (0) & AP (0)] performed the best in the ROI-based multimodal radiomics model with AUC and ACC values of 0.758 and 0.616, respectively, in the testing cohort. In the corresponding dual-region radiomics model created by combined modeling, the ROI’s tumor and peritumor (20 mm) based on multimodal radiomics model [T1WI (20) & PVP (20)  & AP (20)] performed better in predicting MVI risk grading with AUC and ACC values of 0.778 and 0.636, respectively, in the testing cohort.

**Table 4:** Multimodal radiomics models based on different ROIs.

Modality	ROI	Training group		Testing group	
		AUC	ACC	AUC	ACC
T1WI+AP+PVP	Tumor	0.939	0.806	0.758	0.616
	Tumor & Margin (10)	0.947	0.818	0.743	0.606
	Tumor & Margin (20)	0.953	0.838	0.778	0.636

#### 3.2.3 Comparison of Clinical Radiomics Models and Optimal Radiomics Models

**Figure 4:**
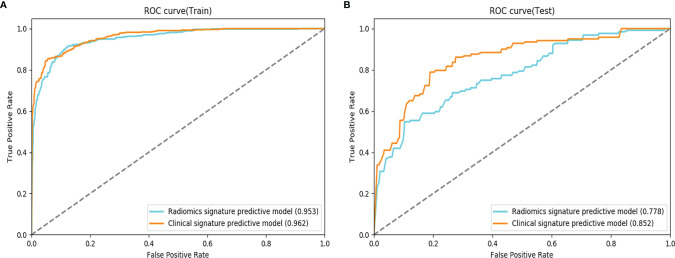
Comparison of receiver operating characteristic (ROC) curves for the prediction of microvascular invasion. ROC curves of the radiomics signature predictive model and the clinical signature predictive model, which combines the fusion radiomics signature and clinicoradiological factors in the training **(A)** and testing **(B)** datasets.

The clinical radiomics model [T1WI (20) & AP (20) & PVP (20)] was more effective than the corresponding multimodal radiomics predictive model in the testing cohort (AUCs: 0.852 vs 0.778; ACCs: 0.747 vs. 0.636) (**Figure 4**).

### 3.3 Survival Analysis

**Figure 5:**
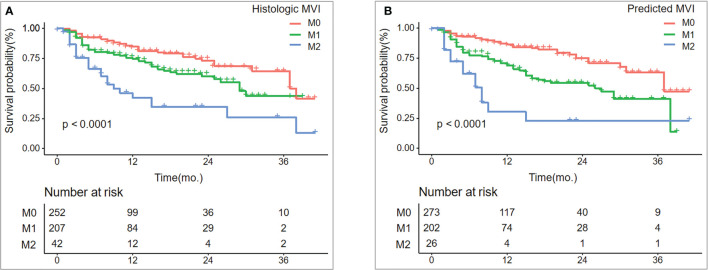
Recurrence-free survival (RFS) between histologic MVI and predicted MVI.RFS **(A, B)** curves were scaled by histologic MVI status and final model-predicted MVI risk grades with Kaplan Meier analysis.

As of December 31, 2020, 501 patients had completed tumor recurrence-free follow-up. The overall recurrence rate was 24.35% (122/501). The median RFS was 38 months for patients in the M0 group, 29 months in the M1 group, and nine months in the M2 group (log-rank test, p < 0.001). Similar results were observed in the prediction model: median RFS was 37 months for patients in M0, 27 months in M1, and eight months in M2 (log-rank test, p < 0.001) (**Figure 5**).

## 4 Discussion

In this study, we analyzed the radiomics signatures of enhanced MRI for tumor and peritumor to determine the impact of dual-region radiomics signatures of tumor and peritumor on different phases of predicting MVI risk grading. We also explored the efficacy of a combined model for clinical factors, imaging features, and radiomics signatures in predicting MVI risk grading. Our results indicated that the impact of dual-region radiomics signatures for tumor and peritumor on predicting MVI risk grades varied at different phases. The established clinical multimodal radiomics model predicted MVI grades with 83.3% and 74.7% accuracy, in the training and testing cohorts, respectively.

The radiomics model that used tumor and peritumor (10 mm or 20 mm) was superior to the radiomics model that was based only on the tumor in predicting MVI risk grades on arterial and portal images. Nebbia et al. (20) found the similar results. The dual-region radiomics signatures of the tumor and peritumor in the late arterial and portal phases were more beneficial in predicting MVI than single-region radiomics signatures. The occurrence of MVI is a complex biological process involving many factors. According to the related studies of Zhou (33) and Wan (34), the activation of epithelial-mesenchymal transformation (EMT) transcription may be an important pathogenic mechanism of MVI in HCC. When EMT transcription is activated, intercellular adhesion proteins such as E-Cadherin are down-regulated and EMT markers such as N-Cadherin and vimentin are increased, which may induce HCC dedifferentiation and increase tumor invasiveness, which leads to the occurrence of MVI (35). MVI is commonly found in the portal vein branches of the liver tissue adjacent to the tumor (29), which is related to the fact that portal vein is the main outflow vessel of liver cancer. When the tumor embolus invades the tiny portal vein, it will cause small branch occlusion and reduce the blood flow of the portal vein around the tumor, giving rise to compensatory peri-tumor hyperperfusion (30, 36). Peritumoral hemodynamic changes can lead to different imaging findings, such as abnormal peritumoral enhancement in arterial phase and low signal intensity in hepatobiliary phase, which are risk factors for MVI. These shows that the peritumoral area of HCC plays an important role in the diagnosis of MVI. This may explain why the combination of tumor and peritumoral radiomics features on AP or PVP images are more conducive to the prediction of MVI risk grade. Feng et al. illustrated that the ROI based on hepatobiliary-phase radiomics model (tumor and peritumor; 10 mm) outperformed the radiomics model based only on the tumor in predicting negative or positive MVI in HCC (15). However, in this study, the ability of the dual-region radiomics model (ROI based on the tumor and the peritumor; 10 mm and 20 mm) to predict MVI risk grades was not better than that of the single region radiomics model based on tumor only. This is because the rate of Gd-EOB-DTPA uptake by hepatocytes is correlated with liver function. Thus, patients with impaired liver function should be reasonably delayed in hepatobiliary-phase scans (37).

This study also found that fusing multimodal radiomics signatures improved the ability of the radiomics model to predict MVI risk grades. Ma et al. (19) made similar conclusions using a multimodal radiomics model, which combined radiomics signatures from enhanced CT arterial, portal venous, and delayed phases. The multimodal radiomics model exhibited better performance than the corresponding unimodal radiomics model in predicting the presence or absence of MVI. This indicated that the inclusion of radiomics signatures from different modalities could improve MVI predictors for various aspects of the tumor. However, the predictive efficiency of the model did not always improve with the incorporation of more radiomics signatures of different modalities. Therefore, this study demonstrated that the number of modalities for radiomics analysis and the diagnostic performance of models are not positively correlated. According to Zhang et al., the AUC values for their radiomics model, which incorporated AP, PVP, and DP in the training and validation cohorts, were 0.784 and 0.82, respectively. However, the radiomics model fusing six sequences of T1WI, T2WI, DWI, AP, PVP, and DP had AUC values of 0.778 and 0.803 in the training and validation cohorts, respectively (26). Merging radiomics signatures for numerous models may exclude radiomics signatures that respond to different traits of the tumor and are meaningful for MVI prediction due to low correlation in the signature screening. Therefore, the performance of the model decreased. Accordingly, further research into the optimal modalities combination for imaging radiomics analysis in MVI prediction is recommended.

The importance of combining different aspects such as laboratory tests, imaging features, and radiomics signatures in predicting MVI was proven in this study. The model that incorporated clinical and radiological signatures such as serum AFP level, cirrhosis, PT, shape, hepatobiliary-phase peritumor hypointensty, and intra-tumoral hemorrhage had higher AUC values than the radiomics model, which corroborates the results of Xu’s research. Xu et al. combined AST, AFP, tumor margins, growth pattern, envelope, peritumor enhancement, and radiomics score to create a nomogram with AUC values of 0.909 and 0.889 in the training and validation cohorts, respectively. In contrast, the radiomics model had AUC values of 0.841 and 0.819 in the training and validation cohorts, respectively (24). Yang et al. also found similar results (25). This suggests that combining the three aspects can improve the ability of models to predict MVI.

The final prediction model established in this study could provide effective prognostic stratification for HCC patients. Tanaka et al. found significant differences in the prognosis of HCC patients with different degrees of MVI progression (38). Therefore, preoperative prediction of MVI grades could more accurately assess the severity of MVI and prognosis of HCC patients than the prediction of presence or absence of MVI, providing clinicians with more beneficial information. In this study, the recurrence-free survival time predicted by the clinical multimodal radiomics model was significantly different among patients with different MVI gradings (*p* < 0.001). Moreover, HCC patients in the M2 group had a significantly shorter recurrence-free survival time than those in the M1 and M0 groups. This finding suggested that our model could assist clinicians in assessing the prognosis of HCC patients preoperatively and providing more personalized treatments.

## 5 Limitations

Although, our clinical radiomics model can be used as a preoperative predictor of MVI risk grading, it has the following limitations. First, all of the MRI images were generated by the same machine at the same hospital. Although this may reduce certain confounding effects, external validation, which could have generated more data from multiple centers, was missing. Future research should include multi-center data to perform independent external validation to confirm the predictive validity of the model. Second, a lack of consistency in the clarity of tumor boundaries on different simultaneous images may have resulted in less accurate tumor ROI segmentation. Therefore, the MRI scanning technique should be updated to obtain clearer tumor contours and establish an automatic segmentation model for HCC to reduce the segmentation discrepancy of ROI. Lastly, the unbalanced data volume between M0, M1, and M2 groups may have affected the predictive performance of the model. Thus, a more considerable amount of data is required to balance differences between the various groups.

## 6 Conclusion

In summary, the radiomics signatures of the dual regions for tumor and peritumor on different phases have diverse effects on the prediction of MVI risk grades. The radiomics signatures of the dual regions for tumor and peritumor on AP and PVP images are of merit to predict MVI. Our final preoperative prediction model can assist clinicians in the preoperative diagnosis of HCC for MVI risk grading and prognostic assessment.

## Data Availability Statement

The raw data supporting the conclusions of this article will be made available by author JW at wangjian@aifmri.com, with reasonable request.

## Ethics Statement 

The studies involving human participants were reviewed and approved by The ethical review committee of the First Affiliated Hospital of the Army Medical University. Written informed consent for participation was not required for this study in accordance with the national legislation and the institutional requirements.

## Author Contributions

All authors made substantial contributions to the conception and design of the study, acquisition of data, analysis and interpretation of data, drafting the article, and revising it critically for important intellectual content. All authors contributed to the article and approved the submitted version.

## Funding

This study was supported by the National Key Research and Development Program of China (No. 2016YFC0107101).

## Conflict of Interest

Author ML and Author SL were employed by Shanghai United Imaging Intelligence Co., Ltd.

The remaining authors declare that the research was conducted in the absence of any commercial or financial relationships that could be construed as a potential conflict of interest.

## Publisher’s Note

All claims expressed in this article are solely those of the authors and do not necessarily represent those of their affiliated organizations, or those of the publisher, the editors and the reviewers. Any product that may be evaluated in this article, or claim that may be made by its manufacturer, is not guaranteed or endorsed by the publisher.
